# Robotic monitoring of Alpine screes: a dataset from the EU Natura2000 habitat 8110 in the Italian Alps

**DOI:** 10.1038/s41597-023-02764-1

**Published:** 2023-12-01

**Authors:** Franco Angelini, Mathew J. Pollayil, Barbara Valle, Marina Serena Borgatti, Marco Caccianiga, Manolo Garabini

**Affiliations:** 1 Centro di Ricerca ‘‘Enrico Piaggio”, and Dipartimento di Ingegneria dell’Informazione, Università di Pisa, Largo Lucio Lazzarino 1, 56122 Pisa, Italy; 2 Università degli Studi di Milano, Department of Biosciences, Via Celoria 26, 20133 Milano, Italy; 3 Department of Life Sciences, Università degli Studi di Siena, Via A. Moro 2, 53100 Siena, Italy; 4 NBFC, National Biodiversity Future Center, Palermo, Italy

**Keywords:** Electrical and electronic engineering, Biodiversity, Community ecology

## Abstract

The surveying of European Union (EU) Annex I habitat “8110 - Siliceous scree of the montane to snow levels (Androsacetalia alpinae and Galeopsietalia ladani)” is generally executed by humans. However, robots could increase human monitoring capabilities. To this end, we collected information on this habitat employing the quadrupedal robot ANYmal C. These data include videos of eight different typical or early warning species. Additionally, data on four relevés are provided. These consist, for instance, of the robot state, and videos and pictures collected to evaluate the habitat conservation status. The aim of this dataset is to help researchers in a variety of fields. For instance, information on plant species collected by the robot can be utilized to develop new procedures and new metrics to assess the habitat conservation status or to train neural networks for plant classification. On the other hand, engineers can use robot state information to validate their algorithms. This database is publicly available in the provided Zenodo repository.

## Background & Summary

Natura 2000 Network is one of the world’s largest networks of protected lands^1–3^. This was created by the European Union (EU) through the European Directive 92/43/EC “Habitats”^4^ with the goal of fighting against biodiversity loss. Among the possible actions to achieve this goal, one is the regular evaluation of the conservation status of the habitats and species included in the Annexes to the “Habitats” Directive^5–7^.

This paper focuses on EU Annex I habitat “8110 - Siliceous scree of the montane to snow levels (Androsacetalia alpinae and Galeopsietalia ladani)”, which is a fragmented habitat linked to peculiar environmental conditions and whose conservation status has been reported as “unfavorable” for the Alpine region of Italy within the official periodical reporting of the conservation status of the EU habitats^8^. The conservation status of these habitats is evaluated through sampling field relevés, which focus on a few key indicators such as vegetation cover, size and distance among patches, debris mobility, and the presence of typical species (TS)^9^. For instance, the occurrences of species linked to mineral, coarse and often unstable substrate, such as *Geum reptans*, *Cerastium uniflorum*, *Ranunculus glacialis*, *Leucanthemopsis alpina*, indicate a good conservation status of the scree habitat^10^. Conversely, the presence of early warning species (EWS) may suggest that the habitat is changing due to a stabilization of the debris or to eutrophication^9^. Field relevés are usually performed by expert plant scientists following national^9^ and international standards; specifically, vegetation sampling follows the phytosociological method^11^. In the case of habitat 8110, a sampled field relevé with a minimum homogeneous area of 16–20 m^2^ is required to measure vegetation cover and to record the presence of typical or early warning species^9,12^. However, this process needs to be repeated in many different locations over time, leading to a not negligible effort by expert plant scientists. The Natural Intelligence project (https://www.nih2020.eu/) aims to improve human monitoring abilities through the application of robotic technologies. For instance, having multiple robots acquiring relevé data under the supervision of plant scientists could increase the area botanists can cover in the same amount of time. We employ legged robots in particular to collect data on the habitat in a manner that closely resembles surveys sampling field relevé recommended by the monitoring guidelines^4,9,11^. The decision to use this kind of robot involves a trade-off between mobility and battery life^13^.

**Fig. 1:**
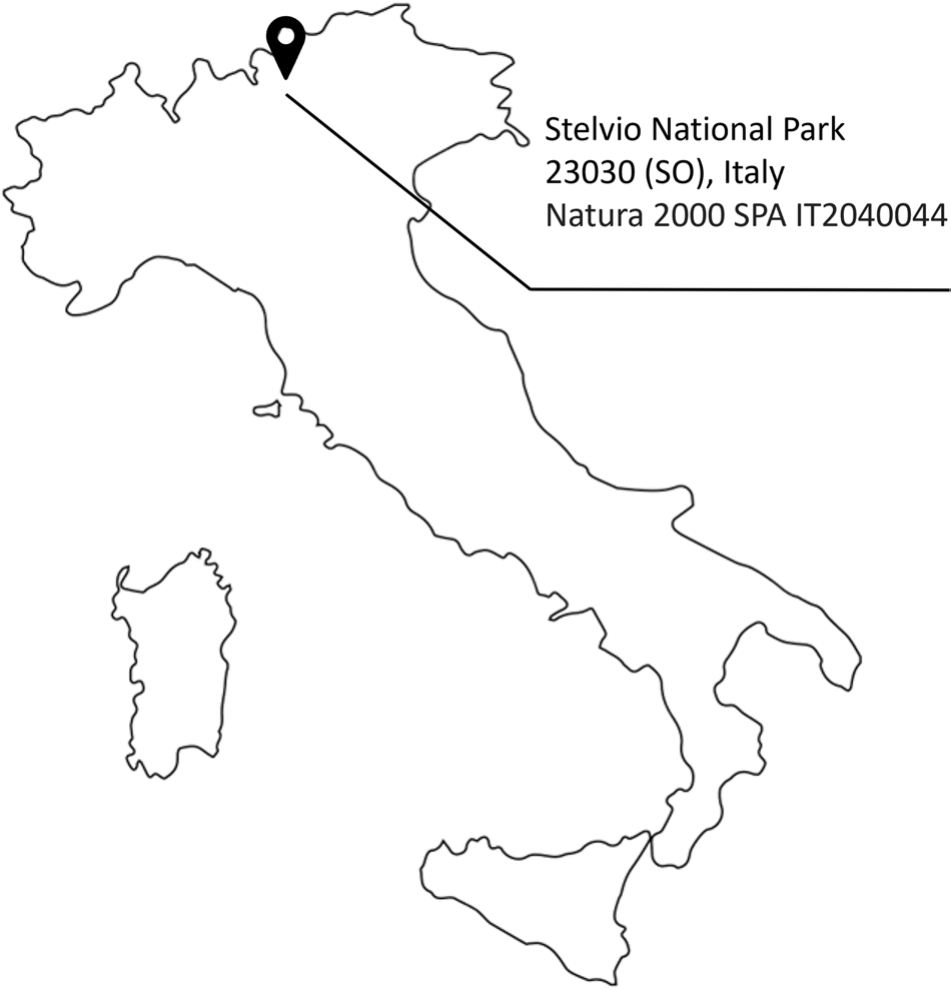
Location of the campaign for data collection.

**Fig. 2:**
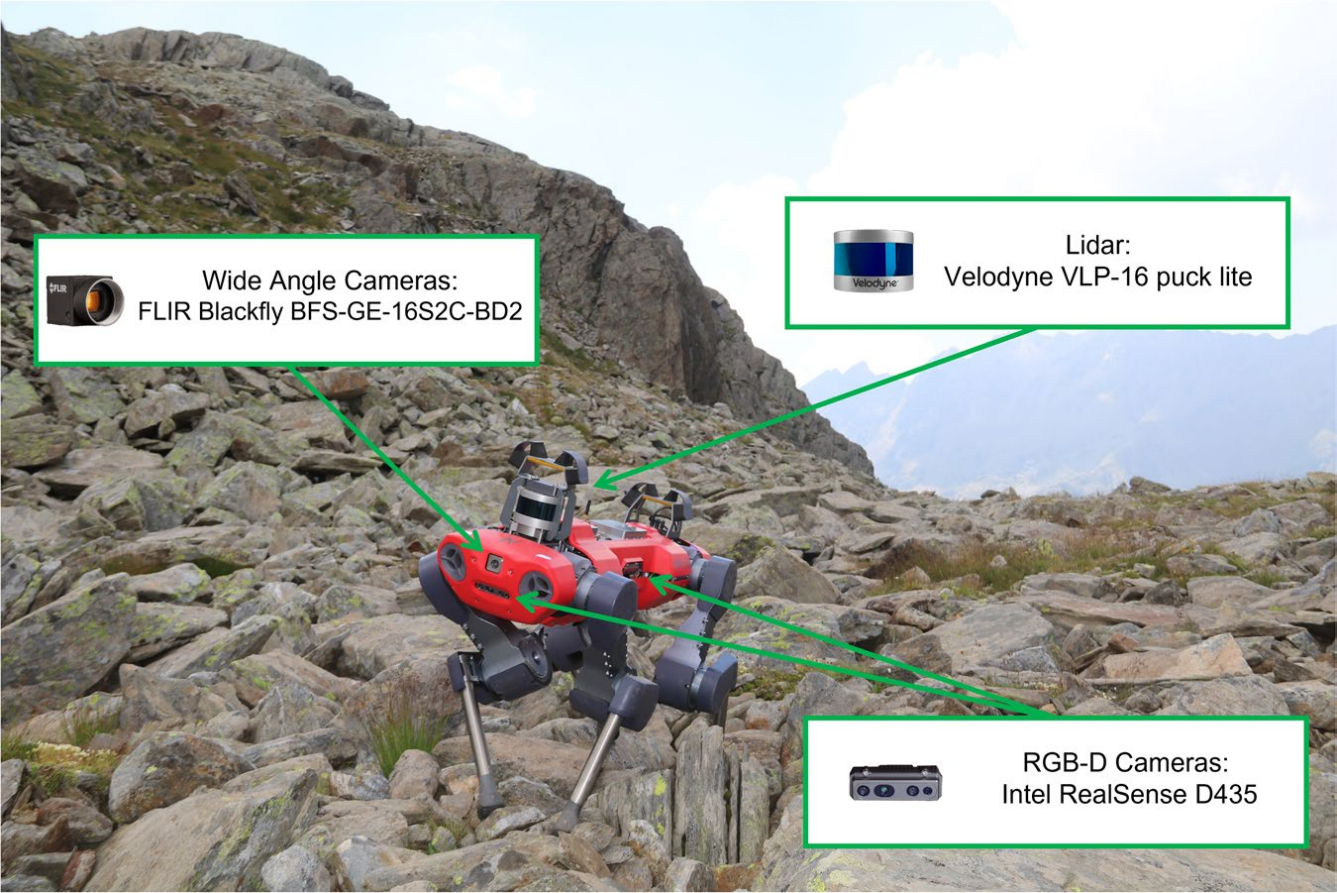
ANYmal C robot gathering data in the habitat 8110. The robot is equipped with different sensors for data acquisition: four Intel RealSense D435 RGB-D cameras, two FLIR Blackfly BFS-GE-16S2C-BD2 wide angle cameras, and one Velodyne VLP-16 puck lite.

This dataset includes data on the monitoring of habitat 8110. The location of the data gathering is the Stelvio National Park, located inside the Natura 2000 SPA IT2040044, Italy (Fig. 1). The monitoring campaign has been performed by a team of both plant scientists and robotic engineers, employing the quadrupedal robot ANYmal C^14^ (Fig. 2). Information within the dataset can be classified into two categories. The first one is a compilation of videos taken by the robot of eight indicator plant species (TS and EWS). In the second section, we report data pertaining to the autonomous monitoring missions. A similar dataset for grassland habitat was already presented in^15^.

The goal of this dataset is to provide data to researchers working in a broad variety of fields. For instance, robot state information and point clouds can be exploited by robotic engineers to benchmark their methodologies. Computer scientists could use plant video and pictures acquired by the robot to design new algorithms for plant identification and classification. The same data could be used by plant scientists to assess the habitat’s conservation status. Finally, this Data Descriptor could drive the botanic community to propose new kinds of relevé exploiting the robotic assistance.

## Methods

A team made up of plant scientists and robotic engineers collected the data in Valfurva, 23030 (SO), Italy, within the Stelvio National Park, located inside the Natura 2000 SPA IT2040044 (Fig. 1). The data collection took place between the 19th and 21st of July, 2022, because in high altitude habitats, this is the best period for the phenology (time of full blooming and development) of vascular plants. Figure 2 depicts the system and the sensors used for data gathering. The former is the quadrupedal robot ANYmal C^14^ created by ANYbotics AG, which is capable of both teleoperated and autonomous motions. The employed sensors are a laser scanner and cameras. The laser scanner is a LiDAR Velodyne VLP-16 puck lite (https://velodynelidar.com/products/puck-lite/) placed on the back of the robot, and it is used to create a 3D map of the surroundings. Cameras are of two different types: RGB-D and wide cameras. The two FLIR Blackfly BFS-GE-16S2C-BD2 wide-angle cameras are not used for data collection. Conversely, the four Intel RealSense D435 cameras (https://www.intelrealsense.com/depth-camera-d435/) are used to record 30 fps movies and/or full HD RGB photos. These cameras are positioned on each side of the system. Through the robot’s ROS interface, data about the robot’s current status is gathered and saved as ROS bag files. Please refer to the official ROS bag reference (http://wiki.ros.org/rosbag) for additional information about this specific file type.

The dataset consists of two distinct pieces of data: (i) information about typical and early warning species, and (ii) information about monitoring missions. The description of the acquisition methodologies follows.

### Typical and early warning species data

**Fig. 3:**
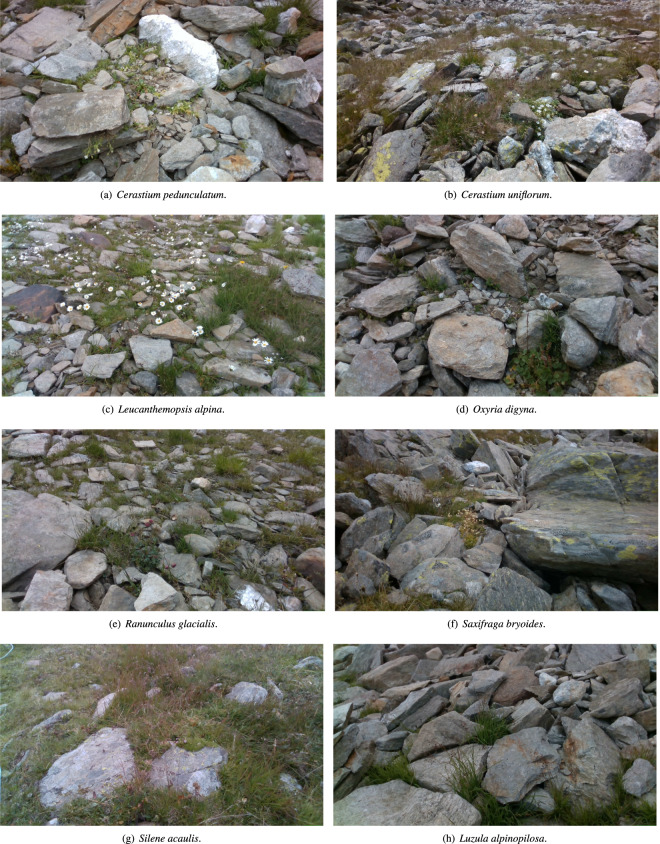
Frames of the videos taken by the ANYmal C robot. (**a**–**g**) are typical species (TS), while (**h**) is an early warning species (EWS).

The first section of the dataset collects videos acquired by the ANYmal C robot of eight indicator species. These are either TS of the habitat 8110 or EWS. The TS were selected among those included in the Italian Interpretation Manual of the 92/43/EEC Directive Habitats^16^ and are: *Cerastium pedunculatum* Gaudin, *Cerastium uniflorum* Clairv., *Leucanthemopsis alpina* (L.) Heywood, *Oxyria digyna* (L.) Hill, *Ranunculus glacialis* L., *Saxifraga bryoides* L., *Silene acaulis* (L.) Jacq. while only one EWS, i.e., *Luzula alpinopilosa* (Chaix) Breistr., has been recorded. The latter is considered an early warning for habitat 8110 because although normally occurring in this habitat, its increase indicates the onset of communities linked to stable substrate conditions, ultimately leading to grassland habitats^17^. The selection of this restricted number of TS and EWS was performed following primarily a botanical criterion (e.g. their higher frequency and their fidelity for the selected habitat), but also considering plant species whose identification could be performed without the sampling of specimens but only by sight. For the nomenclature of plant species, we followed the World Flora Online portal^18^. The first step of the data acquisition was the identification of the indicator species. This has been conducted by a qualified plant scientist who relied on the Italian flora manual^19^. After the species recognition, the robot was placed in front of the selected plant instance by a roboticist through teleoperation, and the video acquisition process was manually launched. For each indicator species, we recorded 6 videos of at least 900 frames each. The items of this section of the dataset are summarized in Table 1. Within each video is present at least one of the indicator species, but they may also show members of other species. An example of the data acquired by the robot for each indicator species is reported in Fig. 3.

**Table 1:** Name and type of the selected eight indicator species of the habitat 8110 and number of recorded videos for each species.

Name	Type	# videos
*Cerastium pedunculatum* Gaudin	Typical species	6
*Cerastium uniflorum* Clairv.	Typical species	6
*Leucanthemopsis alpina* (L.) Heywood	Typical species	6
*Oxyria digyna* (L.) Hill	Typical species	6
*Ranunculus glacialis* L.	Typical species	6
*Saxifraga bryoides* L.	Typical species	6
*Silene acaulis* (L.) Jacq.	Typical species	6
*Luzula alpinopilosa* (Chaix) Breistr.	Early warning species	6

### Monitoring mission data

The second section of the database collects data about the monitoring missions. These follow the same protocols employed by plant scientists during field relevés and recommended by the monitoring guidelines^4,9,11^. First, survey areas are selected following a stratified sampling consisting in finding homogeneous vegetation patches on the mountain side on the basis of station condition, physiognomy and dominant species and selecting the habitat of interest and, within its distribution, placing a random plot^20^. Then, before starting each plot, the date and weather information are noted. This information can be useful to estimate the sun brightness. At the same time, GPS location is recorded using a Garmin GPSMap 64 s sensor with an accuracy of at least 3 m. Georeferencing is crucial for comparing results with prior or future surveys.

Each plot is composed of two steps. In the first one, a qualified engineer teleoperates the robot and moves it around the selected area. During this procedure, the robot employs the LiDAR sensor to generate a 3D map of the surroundings, i.e., to create the environment’s point cloud. The goal of this map is twofold. First, it is necessary to enable the robot autonomous locomotion. Then, it can be useful to determine habitat information, e.g., debris size.

**Fig. 4:**
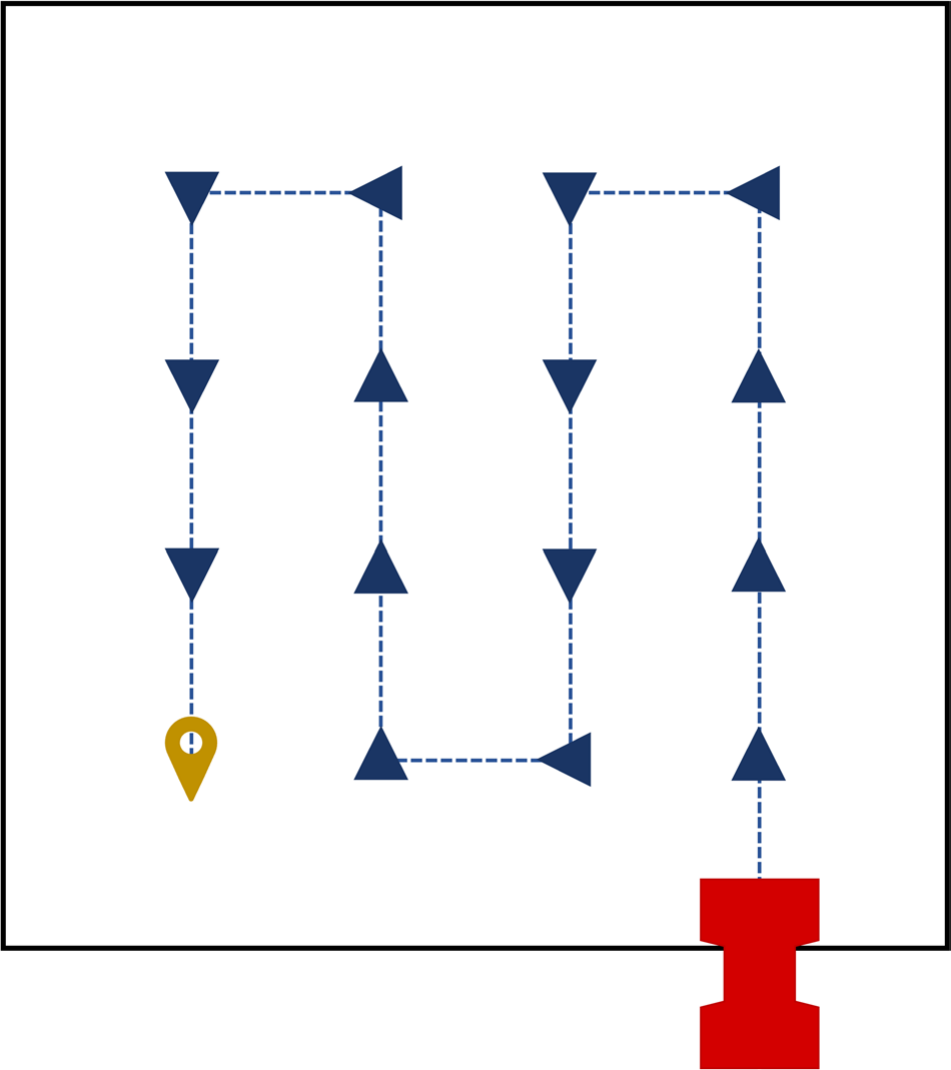
Example of autonomous monitoring mission execution. The red icon indicates the robot, which starts from the bottom right corner of the area to-be-surveyed. The robot motion is depicted through the blue arrows and the blue dashed lines. Arrows indicate waypoints: when the robot reaches a waypoint, it stops and take pictures with all cameras. The final position of the monitoring mission is depicted whit a yellow pin. The robot status information and videos with all cameras are stored during the whole mission execution.

The second step is the autonomous survey performed by the ANYmal C robot. The survey covers the selected surface, which is usually 25 m^2^^9^. To acquire the data, the robot follows a set of waypoints placed on a grid and distanced 1 m one from the other. Starting from the bottom right waypoint, the robot exploits the 3D map to autonomously walk from one waypoint to the other with a square wave motion. As an example, Fig. 4 shows a 4 × 4 grid to cover a 25 m^2^ area.

For the whole mission duration, the four RBG-D cameras mounted on the robot record a video. Additionally, at each waypoint, the robot stops and takes images from the RBG-D cameras.

**Fig. 5:**
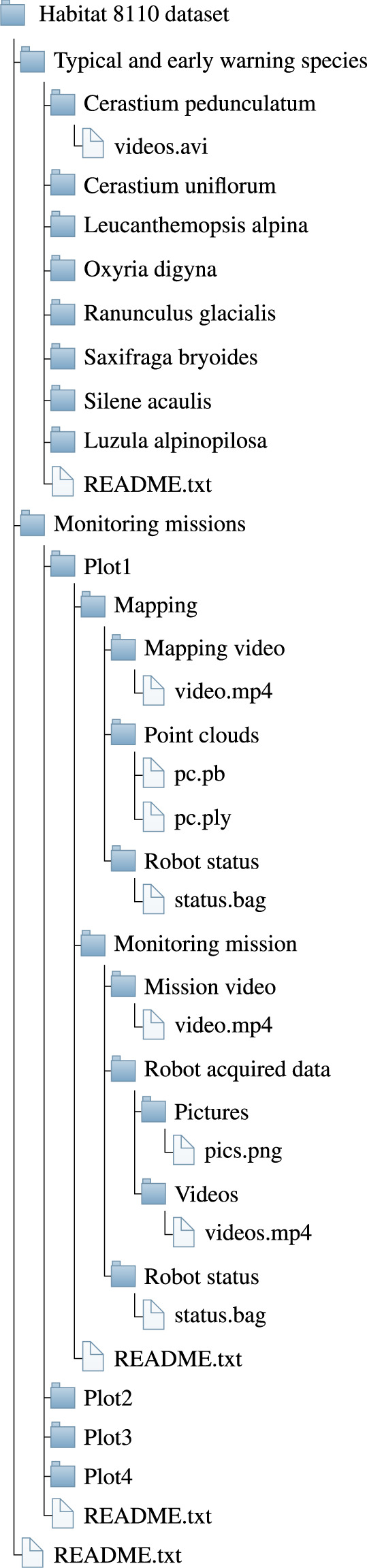
Graphs extracted from ROS bag file of Plot 2. For the sake of readability, only 40 seconds are plotted. Please refer also to the example MATLAB script.

Information about the four executed plots is summarized in Table 2. During both mapping and autonomous missions, we collected also information on the robot status through ROS bag files. These files are related to many ROS topics of the robot control architecture, and they are listed in Table 3. This information includes, for example, joint position, velocities, and acceleration, robot base position, joint torque and current, and battery status. The website of ANYmal Research (https://www.anymal-research.org/) details these topics and the specifications of the robot datastream output. Figure 5 shows an example of the evolution of (part of) the robot status during part of Plot 2. Furthermore, to qualitatively demonstrate the entire procedure, an external video was also recorded during the mapping and the autonomous mission.

**Table 2:** Autonomous monitoring missions date, time, weather information, and location.

Name	Date	Time	Weather	Latitude	Longitude
Plot1	19 July 2022	13:00	Partly cloudy	46°20′30.64″N	10°29′27.47″E
Plot2	20 July 2022	15:08	Partly cloudy	46°20′27.40″N	10°29′24.41″E
Plot3	21 July 2022	14:04	Partly cloudy	46°21′22.58″N	10°30′31.02″E
Plot4	21 July 2022	16:12	Partly cloudy	46°21′21.98″N	10°30′35.40″E

**Table 3:** ROS topic related to the recorded robot information during mapping and autonomous missions.

Topic Name	Description	M	A
/state_estimator/anymal_state	Robot info, e.g., base position [m] and orientation [rad], joint position [rad], velocity [rad/s], acceleration [rad/s^2^], and torque [Nm].	✓	✓
/log/state/current/LF_HAA	Current [A] of the hip adduction/abduction joint of the left fore leg	✓	✓
/log/state/current/LF_HFE	Current [A] of the hip flexion/extension joint of the left fore leg	✓	✓
/log/state/current/LF_KFE	Current [A] of the knee flexion/extension joint of the left fore leg	✓	✓
/log/state/current/LH_HAA	Current [A] of the hip adduction/abduction joint of the left hind leg	✓	✓
/log/state/current/LH_HFE	Current [A] of the hip flexion/extension joint of the left hind leg	✓	✓
/log/state/current/LH_KFE	Current [A] of the knee flexion/extension joint of the left hind leg	✓	✓
/log/state/current/RF_HAA	Current [A] of the hip adduction/abduction joint of the right fore leg	✓	✓
/log/state/current/RF_HFE	Current [A] of the hip flexion/extension joint of the right fore leg	✓	✓
/log/state/current/RF_KFE	Current [A] of the knee flexion/extension joint of the right fore leg	✓	✓
/log/state/current/RH_HAA	Current [A] of the hip adduction/abduction joint of the right hind leg	✓	✓
/log/state/current/RH_HFE	Current [A] of the hip flexion/extension joint of the right hind leg	✓	✓
/log/state/current/RH_KFE	Current [A] of the knee flexion/extension joint of the right hind leg	✓	✓
/pdb/battery_state_ros	Battery information, e.g., charge percentage, voltage [V], current [A].	✓	✓
/tf	Coordinate frames and transformations between them (TFs) (http://wiki.ros.org/tf2)	✓	✓
/path_planning_and_following/navigate_to_goal/result	Info on the success (or failure) of the robot in reaching the desired navigation goal	✗	✓
/path_planning_and_following/trajectory_poses	The planned Cartesian poses (waypoints) tracked by the robot	✗	✓
/path_planning_and_following/active_path	The actual Cartesian path through the waypoints followed by the robot to reach the goal	✗	✓

The **M** and **A** specify which topic has been stored during mapping and autonomous mission, respectively.

## Data Records

The dataset is publicly available on Zenodo^21^ at 10.5281/zenodo.8123332. A code example to visualize the content of the ROS bag files is available on GitHub^22^ and on Zenodo^23^.

**Fig. 6:**
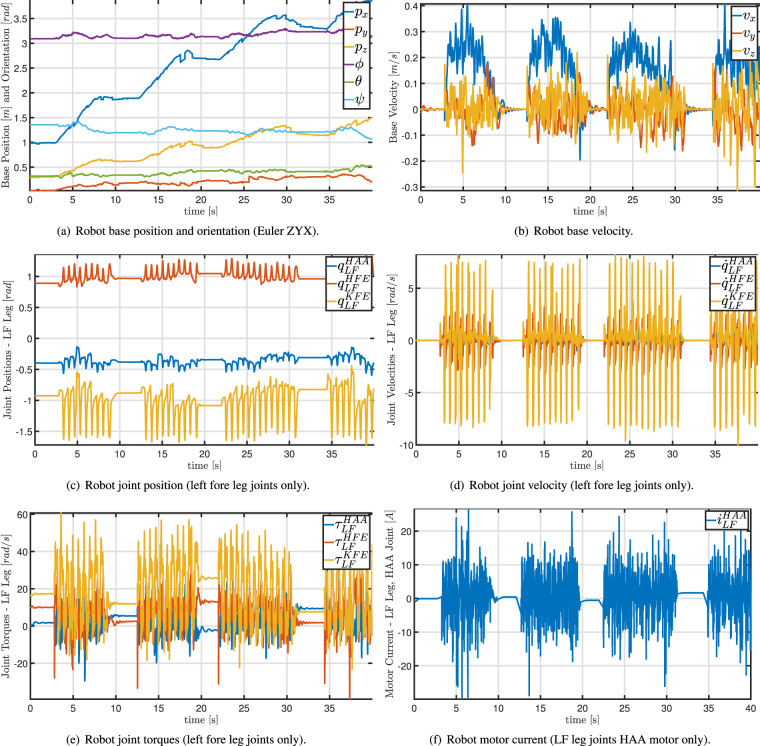
Description of the dataset folder tree.

Figure 6 shows the whole dataset’s tree structure. Each layer of this structure is provided with a README.txt file that briefly describes the layer content. The first layer is divided into the two parts of the dataset: “Typical and early warning species” and “Monitoring missions”. The folder “Typical and early warning species” contains a README.txt file and eight folders, one for each indicator species for habitat 8110. Each indicator species subfolder contains six videos recorded by the ANYmal C robot with at least one instance of that species and eventually of other species. All the entries of this part of the dataset are summarized in Table 1.

The second branch of the dataset, i.e., “Monitoring missions”, organizes the data of the performed relevés into four folders. Each folder contains a “Mapping” and an “Autonomous mission” subfolders, together with a README.txt file. The Mapping subfolders include, in their turn, a README.txt file with the time, date, and weather information and three sub subfolders. These sub subfolders contain the video of the mapping procedure, the acquired point cloud, and the ROS bag files related to the robot state. Each of these bag files is named with the date and time at the start of the recording. Also, the “Autonomous mission” subfolders include a README.txt file with the time, date, and weather information and three sub subfolders. These store a qualitative video of the mission, the robot state information in the form of ROS bag files, and the data collected by the robot. These data are divided into “Pictures” and “Videos” directories, and the name of each file is linked to the position of the camera on the robot body. Specifically, each filename starts with “depth_front”, “depth_left”, “depth_rear”, and “depth_right” depending on the RGB-D camera used to acquire it.

### Data formats

Data reported in the dataset are classified depending on the file format.

#### .jpg

This is a common image file type that can be viewed with a variety of standard image viewer tools. All jpg files in the dataset are pictures taken by the robot during autonomous monitoring missions.

#### .mp4 and .avi

These are common video file formats that may be viewed with many standard multimedia viewer tools. All mp4 files are taken using a reflex digital camera, while the avi files are acquired directly using the RBG-D cameras mounted on the robot. The former files aim to qualitatively show the robot motion during mapping and autonomous missions. The latter files are gathered during autonomous monitoring missions and during the collection of data on the indicator species.

#### .ply and .pb

Point clouds are stored in two formats: pb and ply. The latter, Polygon File Format (ply), is a common format for 3D models. ply files can be visioned with several software tools, for instance, MATLAB (https://www.mathworks.com/products/matlab.html). The former file format, pb, is the default one to store and load point clouds using ROS Gazebo ANYmal research (https://www.anymal-research.org/).

#### .bag

Bag files are the standard file format used by ROS to record data. These are data streams in the form of ROS messages that are directly related to ROS topics (http://wiki.ros.org/rostopic). Table 3 presents the specific list of topics we stored during the mapping and the autonomous monitoring missions. These data are the main information on the robot status. The visualization of these files can be performed in many ways, for instance through several ROS packages or through software for data analysis, such as MATLAB (https://www.mathworks.com/products/matlab.html).

### Example code for data analysis

Data stored in bag files can be visualized using several software tools, such as many ROS packages or MATLAB (https://www.mathworks.com/products/matlab.html). Code 1 shows an example of a MATLAB script to extract robot information from bag files. It is worth highlighting that data provided in this dataset have not been processed through any additional software, and they are provided as they were recorded. Running this example code requires MATLAB 2022a or later and the ROS toolbox (https://www.mathworks.com/products/ros.html). The function *extract_topic_from_bag()* returns a MATLAB structure *msgStructs* containing the data within the specific ROS topic *topic_name* within the ROS bag file *rosbag_file*. Table 3 lists the topics recorded during the performed experiments. The website of ANYmal Research (https://www.anymal-research.org/) presents a more in-depth description of these topics and of the specifications for the datastream output from the ANYmal C robot. However, access to this website is restricted to research institutions that have had their partnership request approved by ANYbotics. Despite this, it should be noted that access to the shared data does not require registering to ANYmal Research, and that partnership with ANYmal Research is only to receive extremely detailed information about the robot. Indeed, the code presented in the next section is enough to read and analyze the ROS bag data.

#### Code 1

Example code to load robot data from ROS bags using MATLAB.

## Technical Validation

Fieldwork quality has been ensured by both the plant scientists i.e., Barbara Valle (BV), Marina Serena Borgatti (MSB), and Marco Caccianiga (MC), and by the robotic engineers, i.e., Franco Angelini (FA), Mathew Jose Pollayil (MJP), and Manolo Garabini (MG). All Authors supervised the data collection and revised the dataset checking for inaccuracies and corrupted files. It is worth mentioning that no alteration through post-processing steps has been applied to the collected data. Indeed, data are shared as raw. The technical validity of the data acquisition is also guaranteed by a set of decisions, whose description is presented in the following subsections.

### Location selection

The selected location is part of the Natura2000 site established in 2009 as ZPS Parco nazionale dello Stelvio (Stelvio National Park) - SPA IT2040044^24^. The SPA comprises 32 Annex I habitats, one of which is a large area of target Annex I habitat 8110, where the presence of TS has already been verified^17^ (please refer to^4,25^ for an explanation of Annex I habitat codes). Official information on the presence and distribution of these habitats may be found in the Park Management plan, edited by Regione Lombardia^26^, or in the EU Standard Data Form of the SACark Management plan, edited by Regione Lombardia^26^. MC, BV and MSB guaranteed *in situ* that the areas selected for data acquisition are typical examples of habitat 8110.

### Date selection

The ideal period to conduct the data gathering is from July to August. However, because the survey region is in a natural setting, the precise decision regarding the ideal time for sampling is largely influenced by seasonal climatic fluctuations in terms of rain and temperature, which have an impact on the phenology. The indicator species’ blooming advances were monitored by MC, BV, and MSB beginning at snowmelt, and the dates 19–21 July were chosen based on the phenology of the indicator species in the research region.

### On field classification of indicator species

Videos contained in the “Typical and early warning species” folder are related to the selected indicator species, which were chosen following international articles^5,16,27,28^. The on-field instances of these indicator species were selected by the involved plant scientists (BV, MSB and MC), which have a sound experience in habitat monitoring and floristic studies in high-elevation scree plant communities^17,27–32^. Additionally, they employed a floristic manual as a guideline^19^.

### Mapping validity

The mapping phase is used to create a digital version of the environment. This is then used by the robot to execute the autonomous mission. For this reason, the mapping accuracy is directly tested by the autonomous mission itself. Indeed, the point cloud validity can be ensured by the fact that the robot is able to localize itself and autonomously move in the scenario.

### Monitoring mission validity

The methodologies employed in the monitoring missions were taken from scientific articles^13^. In order to be compliant with national and international standards^9,11^, these methodologies follow the methodologies employed by plant scientists during field relevé. During the missions, no error message was printed on the screen, ensuring the correct mission execution. Finally, collected data are not post-processed preventing any data adulteration.

### Database validation

Acquired data were put in the database only after their inspection, and corrupted or invalid data were discarded. The inspection step has been performed on each database entry by both teams. MJP also ran test scripts to eventually remove corrupted files. Then, each entry of the robot status directories and of the point clouds directories was inspected by the robotic engineering team to check their correct folder placement. Similarly, each entry of the “Typical and early warning species” folder has been checked by the plant scientists to check their correctness.

## Usage Notes

This database is strongly multi-disciplinary, and for this reason, it can help researchers in a wide variety of fields. For instance, plant videos and pictures can be exploited to develop new procedures or new metrics to assess the habitat conservation status or to train neural networks for plant identification and classification, e.g.,^33^. The robot state information can be used by engineers to validate their locomotion or navigation algorithms. Finally, plant scientists can use these data to assess the habitat conservation status and also to compare them with different plots, e.g., past or future data from the same areas.

## Data Availability

The GitHub page of the Research Center E. Piaggio^22^ contains the MATLAB scripts that were used to extract and visualize data from the ROS bag (.bag) files. This GitHub repository contains also the README files describing these scripts. The same files can also be accessed on Zenodo^23^.
